# Pharmacoeconomic analysis of paliperidone palmitate for treating schizophrenia in Greece

**DOI:** 10.1186/1744-859X-11-18

**Published:** 2012-07-02

**Authors:** Thomas R Einarson, Maria Geitona, Alexandros Chaidemenos, Vasiliki Karpouza, Theodoros Mougiakos, Periklis Paterakis, Dimitrios Ploumpidis, Dionyssios Potamitis-Komis, Roman Zilbershtein, Colin Vicente, Charles Piwko, Panagiotis Kakkavas, Konstantina Paparouni, Rasmus C D Jensen, Michiel E H Hemels

**Affiliations:** 1 Leslie Dan Faculty of Pharmacy, University of Toronto, Toronto, ON M5V 3M8, Canada; 2 Faculty of Social Policy, University of Peloponnese, Korinthos, Greece; 3 Psychiatric Hospital of Athens, Athens, Greece; 4 Psychiatric Hospital of Thessaloniki, Thessaloniki, Greece; 5 401 Military Hospital of Athens, Athens, Greece; 6 Dromokaitio Psychiatric Hospital of Athens, Athens, Greece; 7 Clinical and Social Psychiatry, University of Athens, Athens, Greece; 8 Mental Health Centre of the Psychiatric Clinic of Athens University, Athens, Greece; 9 Private Psychiatric Clinic “Lyrakou”, Athens, Greece; 10 Pivina Consulting Inc, Mississauga, Canada; 11 Medical Advisor Janssen-Cilag Pharmaceutical SACI, Athens, Greece; 12 Janssen-Cilag Pharmaceutical SACI, Athens, Greece; 13 Janssen Cilag, Birkerød, Denmark

**Keywords:** Paliperidone palmitate, Risperidone, Long-acting injectables, Schizophrenia, Pharmacoeconomic analysis, Greece

## Abstract

**Background:**

Patients having chronic schizophrenia with frequent relapses and hospitalizations represent a great challenge, both clinically and financially. Risperidone long-acting injection (RIS-LAI) has been the main LAI atypical antipsychotic treatment in Greece. Paliperidone palmitate (PP-LAI) has recently been approved. It is dosed monthly, as opposed to biweekly for RIS-LAI, but such advantages have not yet been analysed in terms of economic evaluation.

**Purpose:**

To compare costs and outcomes of PP-LAI versus RIS-LAI in Greece.

**Methods:**

A cost-utility analysis was performed using a previously validated decision tree to model clinical pathways and costs over 1 year for stable patients started on either medication. Rates were taken from the literature. A local expert panel provided feedback on treatment patterns. All direct costs incurred by the national healthcare system were obtained from the literature and standard price lists; all were inflated to 2011 costs. Patient outcomes analyzed included average days with stable disease, numbers of hospitalizations, emergency room visits, and quality-adjusted life-years (QALYs).

**Results:**

The total annual healthcare cost with PP-LAI was €3529; patients experienced 325 days in remission and 0.840 QALY; 28% were hospitalized and 15% received emergency room treatment. With RIS-LAI, the cost was €3695, patients experienced 318.6 days in remission and 0.815 QALY; 33% were hospitalized and 17% received emergency room treatment. Thus, PP-LAI dominated RIS-LAI. Results were generally robust in sensitivity analyses with PP-LAI dominating in 74.6% of simulations. Results were sensitive to the price of PP-LAI.

**Conclusions:**

PP-LAI appears to be a cost-effective option for treating chronic schizophrenia in Greece compared with RIS-LAI since it results in savings to the health care system along with better patient outcomes.

## Introduction

Glazer and Ereshefsky [1] were the first to conduct a pharmacoeconomic analysis on patients affected with so-called “revolving door” schizophrenia. The label was adopted to describe persons suffering from chronic disease with multiple relapses, frequent hospitalizations, and problems with adherence to prescribed medications. These patients have many problems and obstacles preventing them from living a normal life. They are also responsible for increased expenditures for healthcare, social services, and the justice system [2].

Antipsychotic drugs can help many revolving door patients to remain in a stable condition; however, a major problem for them is adherence to these prescribed drugs [1,3]. The adherence of patients with schizophrenia is reduced over time. In fact, it has been demonstrated that partial adherence (i.e. missing 25-50% of doses) can reach 50% in 1 year and 75% in 2 years [4]. An important advance in enhancing adherence has been the depot form of these drugs, also referred to as long-acting injectables (LAIs). They have become a mainstay in treatment because of their prolonged effect and consequent prevention of much of the intentional and non-intentional non-adherence that results in treatment failures and hospitalizations [3]. The clinically meaningful superiority of depot medication compared to oral antipsychotic drugs in outpatients with schizophrenia has also been confirmed by the findings of a recent meta-analysis which demonstrated that depot formulations significantly reduced relapses from an average of 33.2% to 21.5% [5].

A further advance has been the development of atypical antipsychotics. They have advantages over the traditional drugs in that they improve both the positive and negative symptoms of the disease [6]. A depot form of atypical antipsychotic was not available until 2002, the first of which was risperidone (RIS-LAI) [7,8]. In a review of the clinical research, Möller concluded that RIS-LAI displayed clinical efficacy and a reasonable degree of tolerability [8]. Moreover, based on the results of a recent multi-centre cohort study across 15 French regions that accounted for 77.6% of the French population in 2005, RIS-LAI use compared to all other LAIs and first or second generation per os antipsychotics was associated with a 34% reduced rate of hospitalization [9]. A clinical disadvantage is that, although clinically effective, it must be administered every two weeks, usually by a specially trained psychiatric nurse or physician [10].

More recently, paliperidone palmitate (PP-LAI) has been developed and approved by the European Medicines Agency [11]. Among the other advantages that it shares with existing drugs, this new product has an added advantage in that it may be administered monthly [12]. PP-LAI is already marketed in several European markets, most often at a higher acquisition price than RIS-LAI.

Although the clinical use of PP-LAI has been investigated in a number of randomized controlled trials [13-17], few economic evaluations have yet been conducted. A search of the international peer reviewed literature revealed one study from the USA that included PP-LAI [18]. However, that study did not use data inputs generated by PP-LAI, but rather they used data from RIS-LAI studies and assumed the two drugs to be exactly equal. Considering differences in dosing regimens, such assumptions and associated cost outcomes may not be valid. Several pharmacoeconomic studies have compared RIS-LAI with other drugs, mainly oral atypicals and traditional depots. In his review of those studies, Haycox found that RIS-LAI was the dominant strategy in all eight different countries, using different analytical models [19].

In Greece, a single pharmacoeconomic study by Geitona and associates [20] was published which focused on paliperidone extended release oral tablets. That study demonstrated that paliperidone was cost-effective over all other oral drugs tested, including risperidone, olanzapine, quetiapine, ziprasidone and aripiprazole. Paliperidone had the lowest overall cost and the highest number of days with stable disease. No other similar studies from Greece could be located.

Given that PP-LAI has a higher acquisition price than RIS-LAI and taking into consideration the scarcity of resources health care systems are faced with, economic evaluation of new technologies is important for decision making purposes. The aim of this paper is to compare costs and outcomes of PP-LAI versus RIS-LAI for the treatment of persons with chronic schizophrenia in Greece.

## Methods

### Patient population

Unlike a clinical trial, patients are not recruited into this research. Rather, a decision model was used to represent the average patient being treated using standard approaches.

Therefore, it is necessary to define the population to whom results would apply. The population of interest consisted of patients having chronic schizophrenia with multiple relapses, frequent hospitalizations, and problems with adherence to prescribed medications. At initiation of the analysis, all patients were stable and treated as outpatients with maintenance doses of their LAIs. For the purposes of this analysis, comorbidities were not considered even though they are common in this population [21].

### Drugs of interest

The primary drug of interest was PP-LAI, which was compared against RIS-LAI. Long term use of PP-LAI has been investigated in a number of randomized controlled trials [13-17]; one of these trials involved a comparison with RIS-LAI [15]. As previously mentioned, the European Medicines Agency has approved PP-LAI for monthly dosing [12], while RIS-LAI is administered every two weeks [10]. It should be noted that at the time of this analysis PP-LAI was not marketed in Greece and there was no local clinical experience.

### Model and base case

**Figure 1:**
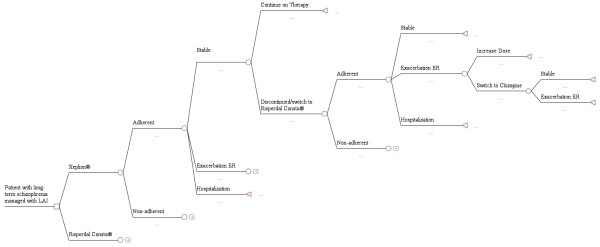
Decision tree model used for the pharmacoeconomic analysis.

Data were modelled for one year using a previously validated decision tree [22], which appears in Figure 1, adapted for use in Greece. An expert panel was recruited and interviewed to provide clinical input describing patterns of patient management in this country. To enter the model, an average patient with chronic relapsing schizophrenia must be an outpatient with stable disease treated with either PP-LAI or RIS-LAI. The patient can be either adherent or non-adherent, according to published rates and expert opinion. Patients can remain stable or can relapse, with treatment either in the emergency room or in hospital for severe cases. Those who cannot tolerate the primary drug or refuse to take it are switched to olanzapine oral tablets. In the event of a subsequent failure on that drug, clozapine oral tablets are prescribed [23,24].

### Clinical inputs

Given the challenge of collecting valid local data for populating the model, which has also been identified in the relevant literature [25], data on resource utilisation, frequency and duration of relapses were mainly extracted from an expert panel consisting of hospital psychiatrists. Other clinical rates and associated data inputs were determined from the literature (Table 1) [10,12-16,23,24,26-40]. The doses of drugs actually administered in long term trials of these drugs were used, rather than the Daily Defined Doses (DDDs) as published by the World Health Organization. DDDs represent the average dose for the drug when used in its most common indication, which does not represent our target population [41]. In the present analysis, the research hypothesis is focused on frequently relapsing patients, necessitating hospitalization and intensive intervention. Therefore, DDDs may underestimate the doses used in the real world when treating these patients, whereas doses from the clinical trials could be considered as better proxy for real world dosing.

**Table 1:** Clinical inputs into the model and sources of information

**Rate**	**RIS-LAI**	**Source**	**PP-LAI**	**Source**
Probabilities				
Adherence	0.823	Olivares [26]	0.872	RIS rate adjusted via Mehnert [27]
Adherent, stable disease	0.763	Calculation [1 - (ER exacerbation rate + hospitalization rate)]	0.803	Calculation [1 - (ER exacerbation rate + hospitalization rate)]
Adherent, exacerbation requiring ER visit	0.071	Ratio of ER vists: hospitalizations Ascher-Svanum [28]	0.059	Ratio of ER vists: hospitalizations Ascher-Svanum [28]
Adherent, hospitalized	0.166	Olivares [29]	0.138	Gopal [14], Hough [13]
Non-adherent, stable	0.140	Kane [30]	0.148	Hough [13]
Non-adherent, exacerbation	0.274	Calculation [1 - (Stable rate + hospitalization rate)]	0.299	Calculation [1 - (Stable rate + hospitalization rate)]
Non-adherent, hospitalized	0.586	Assumption; PP rate adjusted based on calculations by Mehnert & Diels [27]	0.553	Morken [31]
Dosing				
Maintenance dose	40.3 mg biweekly	Fleischhacker [15], Kissling [32], Lee [33], Lindenmayer [34], Olivares [29]	69.3 mg monthly	Gopal[14], Fleischhacker [15]
Dose after relapse	50 mg biweekly*	Risperdal Consta® Approved Summary of Product Characteristics [10] maximum dose	84.9 mg monthly	Gopal [35], Pandina [36], Hough [16], Nasrallah [37], Pandina [38]
Dose after discontinuation	50 mg biweekly*	Risperdal Consta® Approved Summary of Product Characteristics [10] maximum dose	150 mg week 1, 100 mg week 2, then 84.9 mg every 4 weeks	Xeplion® Product monograph [12], Hough [13]
Clozapine maintenance after failing both drugs	450 mg daily	Simonsen[23], Wahlbeck [24]		
Clozapine maximum dose	750 mg daily	Simonsen[23], Wahlbeck [24]		

*ER* emergency room, *PP-LAI* paliperidone palmitate long-acting injection (Xeplion®); *RIS-LAI* risperidone microspheres long-acting injection (Risperdal Consta®)

*A slightly higher average dose of 58.2 mg biweekly was used in clinical trials in patients with acute eacerbations of schizophrenia by Kane [5], Chue [35], and Eerdekens[36]; however, they used a dose of 75 mg mg in some patients, which is not commercially available and which exceeds the now-recommended maximum.

### Cost inputs

Costs were considered from the perspective of the National Health Service of Greece. We included only direct costs of care while indirect costs, such as time lost from work, were excluded (Table 2) [20,42,43]. We did not apply discounting because the analysis had a time horizon of one year. Prices were taken from official bulletins or from the literature, then inflated to 2011 Euros using the Consumer Price Index for Greece [44].

**Table 2:** Cost inputs into the economic model (2011€)

**Resource**	**Item**	**Unit**	**Cost**	**Source**
Drugs	paliperidone palmitate	mg	€ 2.90	calculation^*^
	risperidone microspheres	mg	€ 2.52	calculation^†^
	olanzapine tablets	mg	€ 0.27	calculation^†^
	clozapine tablets	mg	€ 0.0023	calculation^†^
Medical	visit/injection	1 visit	€ 10.12	Geitona [20], Urdahl [42]
Hospital	emergency room	1 visit	€ 50.00	Syriopoulou [43]
	hospital bed acute care	21 days	€ 146 for the first 21 days	DRG tariffs‡
	hospital bed acute care	1 day	€ 45.00/day after 21 days	DRG tariffs‡
	day hospital	1 day	€ 36.86	Geitona [20], Urdahl [42]

^*^Based on available EU prices in the 22 countries used for reference pricing in Greece at the end of September 2011.

†Based on hospital prices published in Price Bulletin 4/8/2011 and IMS Greece market shares July 2011.

‡Calculation based on officially published Ministry of Health Diagnosis Related Group (DRG) tariffs, Government Gazette B 1702/1-8-2011.

### Analysis and outputs

For each drug, we calculated the average cost per patient treated. Patient outcomes analyzed included average days with stable disease, numbers of hospitalizations, emergency room visits, and quality-adjusted life-years (QALYs). To derive utilities (i.e., the quality weights) for this quality adjustment, preference based estimates were obtained from the literature [45-49]. Each of the three primary health states (i.e., stable disease, exacerbation requiring emergency room treatment, and hospitalization) were weighted using the average of the reported utility scores. QALYs were then estimated for each drug by multiplying the amount of time in each health state by the quality value assigned to that health state. Therefore, a cost-utility analysis was conducted, which involves calculating the incremental treatment cost per QALY as the pharmacoeconomic outcome. In addition, a cost-effectiveness analysis was performed in order to assess other important clinical outcomes, such as the number of stable and relapse days as well as rates of hospitalisation and emergency room visits.

Several sensitivity analyses were performed to determine if alterations in clinical or cost inputs would influence outputs. One-way sensitivity analyses were done to identify break-even points, that is, what the values would have to be in order for PP-LAI to cost more than RIS-LAI. We conducted one-way sensitivity analyses on important parameters such as rates of adherence, hospitalization, and emergency room visits as well as drug acquisition costs. Finally, all variables were varied over plausible ranges in a probabilistic sensitivity analysis (also called a Monte Carlo simulation) with 10,000 iterations. That analysis reproduces results for a large group of patients and gives a projection of what average costs and outcomes would be.

## Results

### Cost analysis

The total direct cost to treat one patient over the year was calculated for each drug, as presented in Table 3. Included in those calculations were drugs, medical care (visits) and hospital care, based on the units presented in Table 2. The overall cost to treat patients with PP-LAI was lower than with RIS-LAI by €166, despite having a higher acquisition cost. In the case of PP-LAI, drugs accounted for the largest proportion of the total costs (61%), while hospitalization comprised 30% and medical care the remaining 9%. Costs for RIS-LAI had a similar pattern of distribution, with 56% due to drugs, 33% hospital care and 11% medical care.

**Table 3:** Cost-utility analysis results from comparing paliperidone and risperidone long acting injections for chronic schizophrenia in Greece

**Drug**	Total cost per patient*	**Total QALYs per patient**	**Incremental cost per patient**	**Incremental QALYs per patient**	**Economic conclusion**
PP-LAI	€3,529	0.840	-€166	0.025	dominant
RIS-LAI	€3,695	0.815			dominated

*LAI* long acting injectable, *PP* paliperidone palmitate, *RIS* risperidone microspheres, *QALY* quality adjusted life years.

^*^All costs are in 2011 Euros.

### Cost-utility analysis

The primary pharmacoeconomic analysis was cost-utility, which simultaneously compares costs and QALYs. Along with costs, Table 3 also lists the numbers of QALYs associated with the use of each drug. In the base case, patients treated with PP-LAI have a higher QALY score, meaning that they experience more time with a higher quality of life. Because PP-LAI has a lower cost and a greater number of QALYs, it is considered dominant over RIS-LAI. That means it is the preferred treatment and should be adopted, providing it is affordable.

### Cost-effectiveness analysis

Table 4 displays the other clinical outcomes of interest, which were numbers of stable and relapse days as well as rates of hospitalization and emergency room visits. Patients receiving PP-LAI experienced more days with stable disease and fewer days in relapse. Fewer of them visited the emergency room or were hospitalized. Since PP-LAI has a lower cost and all outcomes were superior, it dominated RIS-LAI in all of these scenarios. These observations confirm that PP-LAI is the preferred strategy to RIS-LAI.

**Table 4:** Cost-effectiveness analysis results

**Drug**	**Stable days per patient**	**Days in relapse per patient**	**Average visits to the emergency room per patient**	**Average number of hospitalizations per patient**	Economic conclusion^*^
PP-LAI	325.0	39.0	0.15	0.28	dominant
RIS-LAI	318.6	45.4	0.17	0.33	dominated

*LAI* long acting injectable, *PP* paliperidone palmitate, *RIS* risperidone microspheres, *QALY* quality adjusted life years.

*Total and incremental costs per patient are onsidered as in Table 3.

### Sensitivity analyses

To test the robustness of the model and its results, an array of different sensitivity analyses were conducted. In one-way sensitivity analyses, the model was insensitive to variations in rates of hospitalization or emergency room visits. That means results would not change and favour RIS-LAI regardless of how many patients were hospitalized or treated in the emergency room. As well, for cost equality between drugs, the adherence rate for PP-LAI would have to decrease by 28.5% (while that of RIS-LAI remained the same), which is not a reasonable scenario.

**Figure 2:**
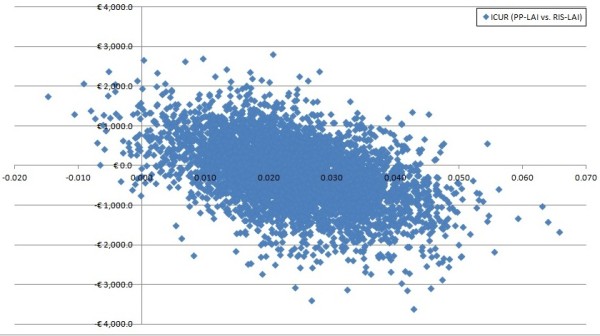
Cost-effectiveness plot of the Incremental Cost Utility Ratio (X-axis = difference in QALYs, Y-axis = difference in costs in 2011 Euros.

In the Monte Carlo synthesis, 74.6% of the 10,000 simulations favoured PP-LAI. Figure 2 depicts the scatterplot of costs versus QALYs. All points below the horizontal line labelled €0.0 represent lower costs for PP-LAI and points to the right of the vertical line represent greater quality of life for patients treated with PP-LAI, as opposed to RIS-LAI.

## Discussion

This pharmacoeconomic analysis aims to assess the clinical and economic value of PP-LAI as a new treatment option in schizophrenia and to support decision making with respect to efficient allocation of resources within the Greek health care setting. Decision analytic modelling was used to estimate and compare the costs and effects of PP-LAI and RIS-LAI in outpatients with chronic schizophrenia within a 1 year time horizon. In this analysis, PP-LAI was the dominant treatment option as it was associated with improved outcomes and a lower average total treatment cost per year.

Overall, the cost of treatment was slightly lower for the healthcare system when PP-LAI was used, despite a higher acquisition cost. It appears that the price of the drug is more than offset by savings accrued from less frequent drug administration and higher adherence rates, as used in our model.

The only other pharmacoeconomic analysis of schizophrenia in Greece was that of Geitona and colleagues who investigated paliperidone extended release tablets [20]. Their calculated cost was just over €7,030 for both paliperidone and risperidone oral tablets, with drugs comprising €1,541 (21.9%) and €1,293 (18.4%) of the totals, respectively. Our overall costs were lower, possibly reflecting the different patient population, i.e.. the analysis by Geitona and colleagues focused on patients with acute exacerbation, or some of it might be attributed to the improved efficacy (i.e., via higher adherence rates) and the reduced hospitalisation rates associated with LAI formulations [50]. In our analysis, drugs accounted for 61% and 56% of the total costs, respectively, which reflect the higher acquisition costs of the LAIs. Nonetheless, the final results were somewhat similar, with paliperidone dominating risperidone.

### Limitations

While reviewing these results it is important to keep in mind the potential limitations of this analysis. An apparent limitation was the fact that local expert panel was used for estimating specific input parameters to the model, namely, those associated with resource utilization in the Greek setting. Although this approach has been followed before in other similar studies [19,20], it could lead to potentially biased estimates. However, in the light of the absence of real life resource utilization data the expert panel could give a picture of the actual clinical setting. Furthermore, in our model we did not include the costs for treating adverse events. One reason was that the drugs are closely related, with paliperidone being a metabolite of risperidone [12]. Therefore, one might expect the efficacy and safety profiles to be quite similar. In fact, that assumption of equal side effect rates was made in a recently published pharmacoeconomic analysis in the USA that included both of these drugs [18]. Geitona and associates [20] did include some of these events, but found that the associated cost was trivial and had no impact on the model. Similar results have been reported by Vera-Llonch and coworkers [51], who estimated the monthly cost associated with side effect management for risperidone and olanzapine. It should be noted that our model captured the discontinuation and switching rates, which are also attributed to adverse events.

### Health policy implications

Efficient resource allocation has become a priority for policy makers across Europe and in Greece in particular. That applies to healthcare as well and to the management of patients with chronic schizophrenia. Health economic studies could provide significant tools for well documented and rational decision making given the scarcity of resources and the increasing control on health care and pharmaceutical expenditure. Pharmacoeconomic analyses, like the one presented, give quantitative estimates of the costs of care and identify the preferred choices for drug treatment. In the case of PP-LAI, its adoption would actually lead to savings for the system, since the overall cost of care would decrease. Both clinicians and managers within the system need to become aware of these analyses and use them to the advantage of both themselves and patients.

## Conclusions

In Greece, PP-LAI should be preferred to RIS-LAI for treating patients with chronic relapsing schizophrenia because it has both clinical and economic advantages. The analysis showed that PP-LAI has a lower overall cost to the health care system and greater clinical benefits in terms of QALYs, days in remission, hospitalizations, and visits to the emergency room for exacerbations of schizophrenia. If adopted, it should result in net savings to the system of €166 per patient treated per year, along with better quality patient care. These findings could be further validated when PP-LAI becomes commercially available in Greece and clinical experience is accumulated. Future research efforts, could focus on conducting economic evaluations based on “real-life” data with respect to clinical outcomes and resource utilization in the local setting, providing this way deeper analysis of the cost-effectiveness of PP-LAI.

## Abbreviations

PP-LAI, Paliperidone palmitate long-acting injectable; QALY, Quality adjusted life-year; RIS-LAI, Risperidone long-acting injectable.

## Competing interests

Thomas Einarson received funding from the sponsor for this research and for the publication of this manuscript. He has also received travel funding to present similar results for Norway at the Latin American ISPOR conference in Mexico City, 2011 and at the European ISPOR Meeting in Madrid, Spain in November 2011. In the past, he has received direct or indirect funding for unrelated work from Amgen Canada, Ferring Canada, Epicept, Generex, Industry Oncology Working Group (Canada), Janssen-Ortho Canada and Novo Nordisk.

Alexandros Chaidemenos, Vassiliki Karpouza, Theodoros Mougiakos, Periklis Paterakis, Dimitrios Ploumpidis, Dionyssios Potamitis and Maria Geitona received honoraria from Janssen-Cilag Greece Pharmaceutical SACI for their participation in the experts’ panel.

Mr. Hemels, Mr. Jensen, and Ms. Paparouni are employees of Janssen.

## Authors’ contributions

TRE was the overall coordinator. TRE, MEHH and RCDJ conceived the study and participated in its design. CV, CP, RZ, KP and MG participated in study design and analysis. AC, DP, VK, TM, DP, PP and PK participated in development of clinical aspects of the model and data collection. All authors were involved in drafting and/or revising of the manuscript. All have read and approved the final manuscript.
